# Nanopore sequencing for identification and characterization of antimicrobial-resistant *Escherichia coli* and *Salmonella* spp. from tilapia and shrimp sold at wet markets in Dhaka, Bangladesh

**DOI:** 10.3389/fmicb.2024.1329620

**Published:** 2024-03-07

**Authors:** Shafiq Rheman, Sabrina Hossain, Md Samun Sarker, Farhana Akter, Laura Khor, Han Ming Gan, Andy Powell, Roderick M. Card, Yaovi Mahuton Gildas Hounmanou, Anders Dalsgaard, Chadag Vishnumurthy Mohan, Zamila Bueaza Bupasha, Mohammed A. Samad, David W. Verner-Jeffreys, Jérôme Delamare-Deboutteville

**Affiliations:** ^1^Laboratory Department of Sustainable Aquaculture, WorldFish, Dhaka, Bangladesh; ^2^Antimicrobial Resistance Action Center (ARAC), Animal Health Research Division, Bangladesh Livestock Research Institute, Savar, Bangladesh; ^3^Department of Sustainable Aquaculture, WorldFish, Penang, Malaysia; ^4^Patriot Biotech Sdn Bhd, Bandar Sunway, Malaysia; ^5^Weymouth Laboratory, Cefas: Centre for Environment Fisheries and Aquaculture Science, Weymouth, United Kingdom; ^6^Veterinary Medicines Directorate FAO Reference Centre for Antimicrobial Resistance, Weybridge, United Kingdom; ^7^Bacteriology Department, Animal Plant Health Agency, Weybridge, United Kingdom; ^8^Department of Veterinary and Animal Sciences, Faculty of Health and Medical Sciences, University of Copenhagen, Frederiksberg, Denmark

**Keywords:** *Salmonella*, *Escherichia coli*, tilapia, shrimp, antimicrobial resistance, whole genome sequencing, food safety, Bangladesh

## Abstract

Wet markets in low-and middle-income countries are often reported to have inadequate sanitation resulting in fecal contamination of sold produce. Consumption of contaminated wet market-sourced foods has been linked to individual illness and disease outbreaks. This pilot study, conducted in two major wet markets in Dhaka city, Bangladesh during a 4-month period in 2021 aimed to assess the occurrence and characteristics of *Escherichia coli* and non-typhoidal *Salmonella* spp. (NTS) from tilapia (*Oreochromis niloticus*) and shrimp (*Penaeus monodon*). Fifty-four individuals of each species were collected. The identity of the bacterial isolates was confirmed by PCR and their susceptibility toward 15 antimicrobials was tested by disk diffusion. The whole genome of 15 *E. coli* and nine *Salmonella* spp. were sequenced using Oxford Nanopore Technology. *E. coli* was present in 60–74% of tilapia muscle tissue and 41–44% of shrimp muscle tissue. *Salmonella* spp. was found in skin (29%) and gills (26%) of tilapia, and occasionally in muscle and intestinal samples of shrimp. The *E. coli* had several Multilocus sequence typing and serotypes and limited antimicrobial resistance (AMR) determinants, such as point mutations on *glpT* and *pmrB*. One *E. coli* (BD17) from tilapia carried resistance genes for beta-lactams, quinolones, and tetracycline. All the *E. coli* belonged to commensal phylogroups B1 and A and showed no Shiga-toxin and other virulence genes, confirming their commensal non-pathogenic status. Among the *Salmonella* isolates, five belonged to Kentucky serovar and had similar AMR genes and phenotypic resistance patterns. Three strains of this serovar were ST198, often associated with human disease, carried the same resistance genes, and were genetically related to strains from the region. The two undetermined sequence types of *S*. Kentucky were distantly related and positioned in a separate phylogenetic clade. Two Brunei serovar isolates, one Augustenborg isolate, and one Hartford isolate showed different resistance profiles. This study revealed high fecal contamination levels in tilapia and shrimp sold at two main wet markets in Dhaka. Together with the occurrence of *Salmonella* spp., including *S*. Kentucky ST198, a well-known human pathogen, these results stress the need to improve hygienic practices and sanitation standards at markets to improve food safety and protect consumer health.

## Introduction

Aquaculture production of tilapia and shrimp in Bangladesh has experienced significant growth in recent years, driven by increasing demands from domestic consumers and export markets (Haque and Belton, 2021; Debnath et al., 2023; El-Sayed and Fitzsimmons, 2023). However, this expansion has raised concerns regarding antimicrobial use (AMU) practices and food safety aspects within the industry. Several studies highlighted the widespread and indiscriminate use of antimicrobials in tilapia and shrimp farms, with a lack of veterinary supervision and adherence to proper dosage and withdrawal periods (Thornber et al., 2020; Dewi et al., 2022). This unregulated AMU in aquaculture settings has been linked to the emergence and spread of antimicrobial resistance (AMR) in bacterial populations, including those present in aquatic environments and seafood products, which can enter the food chain and potentially represent a risk to humans (Lulijwa et al., 2020; Preena et al., 2020; Thornber et al., 2020). Furthermore, studies have reported the presence of AMR bacteria, such as extended-spectrum β-lactamase-producing *Escherichia coli*, *Salmonella* spp., and multidrug-resistant *Vibrio* spp., in aquaculture systems and associated products, posing potential risks to human health (Hamilton et al., 2018; Dewi et al., 2022). Therefore, implementing robust food safety measures from culture ponds to markets is crucial to ensure the sustainability of tilapia and shrimp production and public health safety in Bangladesh.

Wet markets are important points of sales of aquatic food products in Bangladesh and, therefore, play a crucial role in food safety. In Bangladesh, hygiene issues in these markets have always been a major concern. Different factors, including lack of proper sanitation and hygiene practices, inadequate storage and handling, lack of quality control, and extensive use of chemicals contribute to the challenges. Studies have shown that wet markets often have poor hygiene practices and facilities (Alam et al., 2016; Sobuj et al., 2022). In addition, contaminated water may be used to process aquatic produce, leading to the potential contamination of such products by fecal organisms, such as *E. coli* and *Salmonella* spp. (Rocha Rdos et al., 2014; Fernandes et al., 2018). These bacteria can cause foodborne illnesses in humans, and their presence in aquatic products has been suggested as an important source of AMR (Khan et al., 2022; Samad et al., 2023).

The origin of AMR in aquatic products can be attributed to various factors, including AMU in aquaculture, contamination with human and animal fecal matter containing AMR bacteria, and cross-contamination with AMR bacteria during processing and handling (Shikongo-Nambambi et al., 2012; Neela et al., 2015; Watts et al., 2017). The presence of AMR bacteria and associated genes in aquatic products raises food safety concerns, especially since aquatic products are often consumed with minimal cooking or even in their raw state. Resistance genes can subsequently be transferred among bacterial populations in the human intestine, leading to the spread of AMR and potential treatment failure of bacterial infections in humans (Okon et al., 2022).

*E. coli* and *Salmonella* spp. are two major pathogens causing serious infections in humans (Havelaar et al., 2015). *E. coli* causes simple gastrointestinal diseases to more severe diseases, while *Salmonella* spp. are responsible for illnesses, such as typhoid/paratyphoid fever, and non-typhoidal *Salmonella* spp. (NTS) cause food poisoning. The transmission route of *E. coli* and *Salmonella* spp. to humans is mainly via fecally contaminated water and food. *E. coli* and *Salmonella* spp. can develop resistance by selective pressures from antimicrobials and horizontally through the transmission of different mobile elements like plasmids and transposons (Butaye et al., 2006).

In this study, we aimed to develop a pilot genomic surveillance for AMR in *E. coli* and NTS isolated from tilapia and shrimp products purchased at wet markets in Dhaka, Bangladesh. Growth-based methods and PCR were used for bacterial genus identification and their antimicrobial susceptibility was tested by the disk diffusion method. Oxford nanopore sequencing was used to characterize the genomes of *E. coli* and *Salmonella* spp. to confirm their serovars, virulence factors, and the genetic basis of resistance. The findings of the study provide important insight into the occurrence and transmission of antimicrobial-resistant *E. coli* and *Salmonella* from tilapia and shrimp sold at wet markets. These findings can contribute to initiatives aimed at minimizing the risk of transmitting fecal bacterial pathogens and AMR along the food chain.

## Materials and methods

### Selection of fish and shrimp wet markets

For this pilot surveillance study of AMR, tilapia and shrimp specimens were purchased from two wet markets, Karwan Bazar wholesale market (KBW), and Karwan Bazar retail market (KBR), both located in the middle of Dhaka city. These markets are among the largest wholesale and retail wet markets in Bangladesh. Additionally, KBW is the main supplier to a range of different retail markets in Dhaka.

### Collection and processing of fish and shrimp samples

The target species were tilapia (*Oreochromis niloticus*) and tiger shrimp (*Penaeus monodon*), which are commonly sold at the markets. Tilapia, ranging between 250 and 300 g, were mostly sold fresh or live whereas shrimp, 25–30 g each, were displayed on trays occasionally on ice by the traders at the markets. A total of 3 separate visits were made to KBR to collect 27 tilapia. During each visit, nine fresh tilapia were collected from one vendor; however, the vendor changed between the visits. Similarly, four separate visits were made to KBW to collect 27 fresh tilapia. For shrimp sample collection, two visits were made to KBR and two visits were made to KBW to collect 54 shrimp samples (27 from KBR and 27 from KBW). During each sampling, samples were collected from one single vendor; however, vendors were different at every visit. Sample numbers included: KBW: tilapia (*n* = 27), shrimp (*n* = 27); KBR: tilapia (*n* = 27), shrimp (*n* = 27). The number of shrimps purchased from each vendor between the visits was not the same. A total of 18 and 9 shrimp samples were collected during visit 1 and visit 2 at KBW, respectively. In a similar way, the same number of shrimp samples (18 and 9 samples) were collected during visit 1 and visit 2 at KBR market.

Trained personnel wearing sterile plastic gloves collected the samples at the markets, which were individually placed in sterile labeled zipper plastic bags kept in insulated boxes containing ice packs. Samples were transported to Savar, Dhaka, for further processing and analysis in the Animal Health laboratory of the Bangladesh Livestock Research Institute (BLRI). The duration time from the point of sampling at the market until returning to BLRI to start processing the samples was never more than 3 h.

Surface skin swabs, gill, muscle, and intestinal samples were obtained from tilapia using standardized methods. A sterile cotton swab was used to make three long body swipes while twisting the swab to maximize mucus collection along the body (head to tail). The outer surface of the fish was then sterilized by wiping a gauze pad soaked with 70% ethyl alcohol. Using a sterile scalpel and a pair of forceps, the skin was removed and approximately 50 g of muscle and 10 g of gills were collected (in excess) from both sides of the fish. The liver and other organs were carefully removed, and the gut was obtained by cutting it from both extremities from the pyloric caeca to the anus.

The outer surface of the shrimp was disinfected as done for tilapia. Using sterile scissors or scalpel, the intestine from the hepatopancreas to the anus was removed and the content was carefully extruded into a sterile falcon tube. After removing the shell, a meat sample for each shrimp (approximately 15 g) was collected. The different sample types were placed in separate sterile containers or tubes and labeled with unique designation numbers. All collected tissues from each specimen were processed and analyzed separately. Collected tilapia and shrimp specimens were stored at 4°C within two hours upon arrival at the laboratory.

### Bacterial culture and species identification

Each sample type was analyzed for *E. coli* and *Salmonella* spp. All the samples were enriched in sterile trypticase soy broth (TSB, Oxoid, Hampshire, UK) following a 1:10 ratio. From each tilapia, approximately 25 g of muscles and 5 g of gill tissues were used and added to 225 mL and 45 mL of TSB, respectively, in separate stomacher bags. Approximately, 1 g of tilapia gut consisting of a mixture of two pieces of foregut and hindgut was added to 9 mL TSB in a 15 mL Falcon tube and the skin swab was placed into a 15 mL Falcon tube containing 5 mL of TSB. Ten grams of shrimp muscle tissue were added to 90 mL TSB in a stomacher bag and the whole intestine of the shrimp was added into a 15 mL Falcon tube containing 3 mL of TSB. All the samples were incubated for 18 ± 2 h at 37 ± 1°C.

A loopful of enriched broth culture was streaked onto different selective and indicative media as follows. *E. coli* was isolated on eosin methylene blue (EMB; Oxoid, Hampshire, UK) agar plates, which were incubated at 35–37°C for 18–24 h. *Salmonella* spp. was isolated on xylose lysine deoxycholate agar (XLD; Oxoid, Hampshire, UK) plates incubated at 35–37°C for 18–24 h.

Presumptive *E. coli* colonies on EMB and *Salmonella* spp. colonies on XLD agar were streaked onto nutrient agar (NA, Oxoid, Hampshire, UK) to test and ensure purity. The indole test for *E. coli* and the triple sugar iron (TSI) test for *Salmonella* spp. were done for biochemical validation. Isolates were stored at-70°C in 30% glycerol with brain heart infusion (BHI; Oxoid, Hampshire, UK) broth. In case the DNA extraction from the bacterial isolates was not done on the same day, a 10 μL loop full of bacterial suspension for each isolate was preserved/fixed in a 1.5 mL tube containing 500 μL of 100% ethanol with the tubes stored at-20°C for future DNA extraction. *E. coli* ATCC 25922 and *S. enteritidis* ATCC 4931 were used as positive controls when subculturing on selective agar media and for PCR. All selected isolates were Gram-stained following standard procedures. The identity of the presumptive *E. coli* and *Salmonella* spp. was confirmed by PCR (Table 1).

**Table 1:** PCR primers used for the identification of *E. coli* and *Salmonella* spp.

Primer name	Sequence (5′ – 3′)	Amplicon size (bp)	Reference
*E. coli*		166	(Heijnen and Medema, 2006)
*uid*A (F)	TAT GGA ATT TCG CCG ATT TT		
*uid*A (R)	TGT TTG CCT CCC TGC TGC GG		
*Salmonella enterica* spp.		429	(Malkawi, 2003)
ST11	AGC CAA CCA TTG CTA AAT TGG CGC A		
ST15	GGT AGA AAT TCC CAG CGG GTA CTG		

### Antimicrobial susceptibility testing (AST)

AST was done using the disk diffusion method following the Clinical & Laboratory Standards Institute (CLSI) guideline VET01S-Ed5 (CLSI, 2020). Bacterial suspensions were prepared in phosphate-buffered saline solution to a 0.5 McFarland standard and spread onto Mueller Hinton agar (MH; Oxoid, Hampshire, UK) plates. Antimicrobial disks were placed on the surface of the MH agar plates, which were incubated at 35–37°C for 18–24 h. Disks with the following antimicrobials were used: ampicillin (AMP; 10 μg), azithromycin (AZM; 15 μg), chloramphenicol (CHL; 30 μg), cefepime (CEP; 30 μg), cefoxitin (FOX; 30 μg), ceftriaxone (CRO; 30 μg), cefuroxime sodium (CXM; 30 μg), ciprofloxacin (CIP; 5 μg), gentamicin (GEN; 10 μg), levofloxacin (LVX; 5 μg), meropenem (MEM; 10 μg), nalidixic acid (NAL; 30 μg), nitrofurantoin (NIT; 100 μg), norfloxacin (NOR; 10 μg), and trimethoprim-sulfamethoxazole (SXT; 1.25/23.75 μg) (Oxoid, Hampshire, UK). Inhibition zones were measured in millimeters using the scan inhibition zone reader (Interscience Scan 4,000, Interscience, France). Interpretation of the data was done using CLSI breakpoints for all antimicrobials except for nitrofurantoin, of which the zone of inhibition was measured in accordance with the European Committee on Antimicrobial Susceptibility Testing (EUCAST) (CLSI, 2020; EUCAST, 2023). Whole genome sequencing and bioinformatic analyses.

### DNA extraction and purification

A total of 16 *E. coli* and 14 *Salmonella* spp. isolates from tilapia and shrimp samples were selected for whole genome sequencing and further analysis. Bacterial resuspension was pelleted, followed by the removal of supernatant (ethanol) via decantation. DNA extraction was performed as per the method of (Sokolov, 2000) with some modifications. The pellet was resuspended in 500 μL of lysis buffer (50 mM NaCl, 50 mM Tris–HCL pH8, 50 mM EDTA, 2% SDS) and incubated at 60°C for 30 min. Then, 3 μL RNAse A (10 mg/mL) was added to the lysate, followed by incubation at room temperature for 10 min. Salting out was performed via the addition of 333 μL (2/3 vol) saturated NaCl at 4°C for 5 min. The lysate was centrifuged to remove precipitated proteins and lipids and the aqueous layer containing the DNA was transferred to a new tube and mixed with 100 μL of isopropanol via inversion. Fifteen μl of magnetic beads were added and the solution was mixed by inversion, then incubated at room temperature for 10 min to promote the binding of nucleic acid to the beads’ surface. Beads and DNA were separated from the remaining lysate on a magnetic rack and washed twice with 75% ethanol. DNA elution from the bead was performed by the resuspension of the beads with 50 μL of TE buffer followed by incubation at 50°C for 5 min.

A subsample of the extracted gDNA was visualized by 2% agarose gel electrophoresis to assess overall DNA integrity and yield. DNA was purified and size-selected using 0.5X vol of magnetic beads to remove small molecular weight DNA as well as any other carry-over impurities from the initial extraction. The DNA was subsequently measured using a Denovix High sensitivity kit (Denovix, Wilmington, DE, USA) and normalized to approximately 25 ng/μl.

### Nanopore library preparation and sequencing

Libraries were prepared using the following two rapid barcoding sequencing kits. The first kit, SQK-RBK004, was used for low throughput, up to 24 samples per library. Approximately 400 ng of DNA as measured by Qubit was fragmented according to the manufacturer’s instructions (Oxford Nanopore, UK). The barcoded samples were pooled in equal ratios to get a final 400 ng of genomic DNA and purified using SPRI Bead, followed by ligation of the rapid adapter (RAP). The second kit, SQK-RBK110.96, for high throughput, 96 samples per library, used approximately 50 ng of DNA per sample, and then samples were pooled, purified, and ligated with RAP. The final pooled library was sequenced on a Flongle flow cell for at least 12 h to assess index distribution. Base calling and demultiplexing of the fast5 files were both performed with Guppy v.5.0.7. Based on the index percentage, the tagmented products were re-pooled or re-prepped for large-scale sequencing on the MinION flow cell and base calling (super accuracy mode) and demultiplexing performed as before to generate the final fastq files for each of the samples prepared.

### *De novo* assembly

Raw nanopore reads were adapter trimmed with a qscore of 9 or higher using porechop (Wick et al., 2017) and then filtered to retain reads longer than 2,000 bp using NanoFilt v2.6.0 (De Coster et al., 2018). The filtered Nanopore reads were assembled *de novo* using Flye v2.9 (Kolmogorov et al., 2019), followed by one round of polishing with racon (Vaser et al., 2017) and another round of polishing with medaka v1.4.4^1^. Genome assembly statistics were generated using QUAST (Gurevich et al., 2013). Assessment of the genome completeness was done using BUSCO5 (Simão et al., 2015) and identified conserved microbial single-copy genes as listed in the bacteria_odb10 database.

### Bacterial species identification and *Salmonella* serotyping

Whole genome sequence identification was performed using kraken2 to confirm the bacterial species of the sequenced strains (Wood et al., 2019). Out of the 16 *E. coli* and 14 *Salmonella* spp. Isolates from tilapia and shrimp samples that were sequenced, only 15 *E. coli* and 9 *Salmonella* spp. Genomes were further analyzed based on DNA quality control (e.g., high genomic completeness) (Tables 2, 3).

**Table 2:** Occurrence of *Salmonella* spp. in tilapia and shrimp.

	Tilapia		Shrimp	
	KBW*	KBR**	KBW*	KBR**
Skin	2 (7%)	8 (29%)	NA***	NA***
Gill	14 (52%)	7 (26%)	NA***	NA***
Muscle	0 (0%)	0 (0%)	0 (0%)	4 (15%)
Intestine	1 (3%)	0 (0%)	0 (0%)	2 (7%)
Sub-total	16 (14%)	16 (14%)	0 (0%)	6 (11%)
Total	38/324 (11%)			

* KBW, Karwan Bazar wholesale market, ** KBR, Karwan Bazar retail market, *** NA, Not applicable.

**Table 3:** Genomic characterization and antimicrobial resistance of *E. coli*.

Isolate ID	Date of isolation	Sample source	MLST	Serovar	Phylo group	Resistance patterns*	AMR genes**
BD08	19/9/2021	Shrimp/muscle	1,662	H16-O103	B1	AMP(I); CXM(I)	*glpT*; *pmrB*
BD09	19/9/2021	Shrimp/muscle	48	H9-O8	A	AMP(R); CXM(I)	*glpT*; *pmrB*
BD14	19/9/2021	Shrimp/muscle	196	H16-O8/O160	B1	AMP(I); CXM(I)	*glpT*; *pmrB*
BD01	31/10/2021	Shrimp/muscle	3,640	H49	B1	AMP(I); NAL(I); LVX(I); CXM(I)	*glpT*; *pmrB*
BD03	31/10/2021	Shrimp/muscle	155	H19	B1	AMP(R); FOX(R); CXM(I)	*glpT*; *pmrB*
BD13	31/10/2021	Shrimp/muscle	398	H21-O159	A	CXM(I)	*glpT*; *pmrB*
BD23	31/10/2021	Shrimp/muscle	3,501	H16	B1	CXM(R); CRO(I)	*glpT*; *pmrB*
BD12	12/9/2021	Tilapia/skin	8,349	H7-O18	B1	NIT(R); AMP(I); CXM(I)	*glpT*; *pmrB*
BD05	17/10/2021	Tilapia/skin	und	H7-O83	B1	FOX(R); AMP(I); CXM(I)	*glpT*; *pmrB*
BD19	17/10/2021	Tilapia/skin	und	H19-O8	B1	AMP(I); CXM(I)	*glpT*; *pmrB*
BD21	17/10/2021	Tilapia/skin	155	H12-O28ab	B1	-	*glpT*; *pmrB*
BD22	17/10/2021	Tilapia/skin	und	H19-O8	B1	NIT(R); CRO(R); NAL(I), CXM(I)	*glpT*; *pmrB*
BD11	12/9/2021	Tilapia/muscle	164	H9-O49	B1	AMP(R); MEM(R); GEN(R); CXM(I)	*glpT*; *pmrB*
BD17	17/10/2021	Tilapia/muscle	2,165	H7	B1	AMP(R); CXM(I); NAL(I)	*glpT*; *pmrB*; *bla*_TEM-1B_; *qnrS13*; *tet*(A)
BD20	10/10/2021	Tilapia/muscle	und	H26-O8/O80	B1	NIT (R), CIP (I), CXM (I)	*glpT*; *pmrB*

*Antimicrobials; AMP, ampicillin (10 μg); FOX, cefoxitin (30 μg); CRO, ceftriaxone (30 μg); CXM, cefuroxime sodium (30 μg); CIP, ciprofloxacin (5 μg); GEN, gentamicin (10 μg); LVX, levofloxacin (5 μg); MEM, meropenem (10 μg); NAL, nalidixic acid (30 μg); and NIT, nitrofurantoin (100 μg); ** *glpT* (glycerol-3-phosphate) mutations encode resistance to fosfomycin; *pmrB* (polymyxin B) mutations encode resistance to polymyxins; und: undetermined.

The genomes confirmed as *Salmonella* spp. were further analyzed with sistr_cmd and seqsero for confirmation of the serovar (Zhang et al., 2015; Yoshida et al., 2016). Where a discordant serovar was identified by these two tools, the KMER-based taxonomic assignment of kraken2 was used as the third comparison point where the majority was taken to decide on the serovar.

### *In-silico* identification of multilocus sequence typing (MLST), phylogroup analysis, AMR genes and virulence factors

*In-silico* MLST was performed using the PubMLST database^2^ developed by Keith Jolley (Jolley and Maiden, 2010) and the open-source software (mlst tool) used to interrogate the database^3^ (Jolley et al., 2018). AMR genes were determined in the assembled genomes using Resfinder4.1 (Bortolaia et al., 2020) and AMRFinderPlus (Feldgarden et al., 2021). The virulence determinants of *E. coli* were determined using a virulence factor database (Chen et al., 2005) and the curated VirulenceFinder database for *E. coli* (Malberg Tetzschner et al., 2020), while *Salmonella* virulence was determined by looking for their *Salmonella* Pathogenicity Islands (SPIs) (Roer et al., 2016). Moreover, *E. coli* genomes were subjected to phylogroup analysis using pathogenwatch^4^ and clermontyping tools (Beghain et al,. 2018).

### Phylogenetic analysis

*Salmonella* Kentucky was the main serovar determined in the analysis. Strains with this serovar were added to publicly available *S*. Kentucky genomes from migratory birds (Card et al., 2023), humans, and poultry in Bangladesh and its neighboring countries, such as India (human and sesame seeds), Nepal (human), Pakistan (chili), and Myanmar (human) (Supplementary Table S4). The phylogenetic analysis was run using as reference genome *S*. Kentucky strain 201,001,922 (accession number CP028357). For this analysis, single-nucleotide variants were called using Snippy version 4.6.0^6^ under the following parameters: mapping quality of 60, a minimum base quality of 13, a minimum read coverage of 4, and a 75% concordance at a locus. We aligned core genome single-nucleotide variants by using Snippy Core version 4.1.0 for phylogeny inference and detected masked putative recombinogenic regions by using Gubbins version 2.4.1. A maximum-likelihood phylogenetic tree was built using RAxML version/8.2.12 and the generalized time-reversible model with 200 bootstraps. The final tree was rooted on the reference genome CP028357 and visualized with Microreact.^7^

## Results

### Occurrences of *E. coli* and *Salmonella* spp. in tilapia and shrimp

Surface skin swab, gill, muscle, and intestinal samples from tilapia obtained at the KBW and KBR contained *E. coli* as confirmed by PCR in between 30 and 78% of samples analyzed. There was no apparent association between the different sample types and the different visits to the markets. It is noted that despite muscle tissue samples being obtained following disinfection of the skin surface, 60 to 74% of such samples contained *E. coli* indicating cross-contamination during sample processing as meat samples normally would be expected to be sterile.

*E. coli* confirmed by PCR was found in 41 and 44% of muscle tissue and 22 and 33% of intestinal samples from shrimp at the KBW and KBR markets, respectively. There were no apparent differences in findings during the different visits to the markets.

*Salmonella* spp. confirmed by PCR was found in 29% of skin swabs and 26% of gill samples, but not in muscle tissue and intestinal samples, from 27 tilapia fish collected at KBR (Table 2). *Salmonella* spp. was isolated in 7% of skin swabs, 52% of gill samples, and one intestinal sample of 27 tilapia fish analyzed from the KBW. No muscle tissue and intestinal samples from shrimp obtained at KBW contained *Salmonella* spp. as confirmed by PCR whereas *Salmonella* spp. was isolated and confirmed by PCR in 4/27 (15%) muscle tissue and 2/27 (7%) intestinal samples in shrimp obtained at KBR (Table 2).

### Genomic characterization and antimicrobial resistance of *E. coli*

The genomic characterization (i.e., size, MLST, serovar., phylogroup) and antimicrobial resistance patterns and genes of the 15 *E. coli* isolates obtained from shrimp and tilapia samples and that yielded quality genomes are shown in Table 3. The *E. coli* isolates from shrimp had total genome lengths ranging from 4,725,736 to 4,897,945 base pairs, whereas the *E. coli* isolates from tilapia exhibited total genome lengths ranging from 3,968,775 to 5,077,851 base pairs (see further details in Supplementary Table S1). MLST analysis of the isolates from shrimp revealed the presence of 7 sequence types (STs), including 3,640, 3,501, 1,662, 398, 196, 155, and 48. Serovar classification identified different serovars, such as H49, H19, H16-O103, H9-O8, H21-O159, H16-O8/O160, and H16 (Table 3). The tilapia isolates also display several different MLSTs. Serovar classifications included H7, H9-O49, H7-O18, H19-O8, H26-O8/O80, and H12-O28ab (Table 3).

The phylogenetic analysis of isolates from both shrimp and tilapia revealed that they belonged predominantly to the commensal phylogroups B1 and A (Table 3). The commensal nature of these isolates in warm-blooded animals and humans, but not in fish, was further underlined by the absence of major virulence factors displayed by the known *E. coli* pathotypes in the genomes (Supplementary Table S2). As commensal strains, the genome sequences did not contain any clinically important AMR genes except for point mutations on the glycerol-3-phosphate transporter (*glpT*) and the polymyxin resistance gene B (*pmrB*), known to be associated with potential resistance to fosfomycin and colistin, respectively (Table 3). The isolate BD17 from tilapia harbored the resistance genes, *bla*_TEM-1B_, *qnrS13,* and *tet*(A) encoding resistance to beta-lactams, quinolones, and tetracycline, respectively. None of the *E. coli* strains contained any plasmids. This isolate displayed phenotypic resistance to ampicillin and intermediate resistance to nalidixic acid and cefuroxime sodium (Table 3). Several strains showed phenotypic resistance to ampicillin (4 strains) and nitrofurantoin (3 strains), and several strains showed intermediate resistance to some antimicrobial classes, e.g., 13/14 *E. coli* strains were intermediately resistant to cefuroxime sodium. However, no genes associated with such resistance and intermediate resistance were identified (Table 3). One *E. coli* strain was resistant to ceftriaxone. Further details on the phenotypic resistance are provided in Supplementary Table S5.

### Genomic characterization and antimicrobial resistance of *Salmonella* spp.

The genomic characteristics and serovars of the nine *Salmonella* spp. isolates obtained from tilapia samples are shown in Table 4 (see further details in Supplementary Table S1). These strains yielded quality genomes and were isolated between 12 September and 31 December 2021. All strains were *Salmonella enterica* subspecies *enterica*.

**Table 4:** Genomic characterization of *Salmonella enterica* isolates from tilapia sold at wet markets in Bangladesh.

Isolate ID	Date isolation	Sample source	ST	Serovar	SPI*	Plasmids	Resistance patterns***	AMR genes
BD40	12/9/2021	Tilapia/gills	198	Kentucky	C63PI; SPI-1; SPI-2; SPI-3	IncQ1	CIP(R); AMP(R); NAL(R); NOR(R); GEN(R); LVX(R); CXM (I)	*aac(6′)-Iaa*; *bla*_TEM-1B_; *gyrA* (p.S83F); *sul1*; *tet*(A); *aac(3)-Id*; *aadA7; qacE*
BD41	12/9/2021	Tilapia/gills	und	Hartford	C63PI; SPI-1; SPI-2; SPI-5; SPI-13; SPI-14	none	-	*aac(6′)-Iaa*; *fosA7*
BD43	12/9/2021	Tilapia/gills	und	Kentucky	C63PI; SPI-1; SPI-2	IncQ1	CIP(R); AMP(R); NAL(R); NOR(R); GEN(R); CRO(I)	*aac(6′)-Iaa*; *bla*_TEM-1B_; *gyrA*(p.S83F); *sul1*; *tet*(A); *aac(3)-Id*; *aadA7; qacE*
BD25	10/10/2021	Tilapia/gills	und	Augustenborg	C63PI; SPI-1; SPI-2; SPI-3; SPI-13; SPI-14	none	CXM(I)	*aac(6′)-Iaa*; *fosA7*; *parC*:p.T57S
BD35 **	28/11/21	Tilapia/gills	1794	Brunei	C63PI; SPI-1; SPI-2; SPI-3; SPI-13	IncFII	AMP(R); FOX(R); LVX(R); CRO(R)	*aac(6′)-Iaa*
BD37**	28/11/21	Tilapia/gills	und	Brunei	C63PI; SPI-1; SPI-2; SPI-13; SPI-14	IncFII	AMP(R); FOX(R); GEN(R); NAL(I); CIP(I); CXM(I)	*aac(6′)-Iaa*
BD42	12/9/2021	Tilapia/skin	und	Kentucky	SPI-1; SPI-2; SPI-5; SPI-13	IncQ1	CIP(R); AMP(R); NAL(R); FOX(R); GEN(R); LVX(R); CXM(I)	*aac(6′)-Iaa*; *bla*_TEM-1B_; *gyrA*(p.S83F); *sul1*; *tet(A)*; *aac(3)-Id*; *aadA7; qacE*
BD45	12/9/2021	Tilapia/skin	198	Kentucky	C63PI; SPI-1; SPI-2, SPI-3	IncQ1	CIP(R); AMP(R); NAL(R); NOR(R); GEN(R); LVX(R); CXM(I)	*aac(6′)-Iaa*; *bla*_TEM-1B_; *gyrA*(p.S83F); *sul1*; *tet*(A); *aac(3)-Id*; *aadA7; qacE*
BD46	12/9/2021	Tilapia/skin	198	Kentucky	C63PI; SPI-1; SPI-2; SPI-3	IncQ1	CIP(R); AMP(R); NAL(R); NOR(R); GEN(R); LVX(R); CXM(I)	*aac(6′)-Iaa*; *bla*_TEM-1B_; *gyrA* (p.D87Y); *sul1*; *tet*(A); *aac(3)-Id*; *aadA7; qacE*

* SPI: Salmonella Pathogenicity Islands; ** from wholesale market (all others from retail market).

***Antimicrobials; AMP, ampicillin (10 μg); FOX, cefoxitin (30 μg); CRO, ceftriaxone (30 μg); CXM, cefuroxime sodium (30 μg); CIP, ciprofloxacin (5 μg); GEN, gentamicin (10 μg); LVX, levofloxacin (5 μg); NAL, nalidixic acid (30 μg) and NOR, norfloxacin (10 μg). und: undetermined.

Isolate BD25 exhibited a total genome length of 4,774,230 bp and belonged to the Augustenborg serovar and the AMR genes detected were *aac(6′)-Iaa* and *fosA7*, as well as *parC*:p.T57S mutation. The isolate showed intermediate resistance to CXM. Notably, the pathogenicity islands SPI-1, SPI-13, SPI-14, SPI-2, SPI-3, and C63PI were present, while no plasmids were detected.

Isolate BD35, belonging to the Brunei serovar., had a total genome length of 4,832,090 bp. MLST analysis revealed ST1794 and the presence of the *aac(6′)-Iaa* gene encoding resistance to aminoglycosides. The isolate was resistant to AMP, FOX, LVX, and CRO. SPI-1, SPI-13, SPI-2, SPI-3, and C63PI were identified, along with the IncFII plasmid. BD37 had also the Brunei serovar. The aminoglycoside resistance gene *aac(6′)-Iaa* was also detected together with SPI-1, SPI-13, SPI-14, SPI-2, and C63PI. This isolate was resistant to AMP, GEN, and FOX and showed intermediate resistance to other antimicrobials. Both isolates contained the IncFII plasmid.

Isolate BD41 belonged to the Hartford serovar. The AMR genes, *aac(6′)-Iaa* and *fosA7* (fosfomycin) were present, along with C63PI, SPI-1, SPI-13, SPI-14, SPI-2, and SPI-5. No plasmids were observed. The isolate was fully susceptible to all antimicrobials tested.

Isolates BD42, BD40, BD43, BD45, and BD46, were all the Kentucky serovar and exhibited total genome lengths ranging from 4,887,734 to 4,920,732 bp. These isolates shared similar AMR genes including *bla*_TEM-1B_, *gyrA* mutations, *sul1*, *tet*(A), *aac(3)-Id*, and *aadA7*. The isolates showed similar phenotypic resistance patterns, i.e., to AMP, CIP, GEN, LVX, NAL, and NOR. Some isolates did also show intermediate resistance to other antimicrobials (further details on the phenotypic resistance are provided in Supplementary Table S6). SPI-1, SPI-2, and SPI-5 were identified, along with the IncQ1 plasmid. Three isolates were ST198. They all have the K variant of SGI-1 carrying the same resistance genes all on the same contig in each genome. It should be noted that the plasmids were detected based on their replication proteins not by reconstructing the entire plasmids.

**Figure 1:**
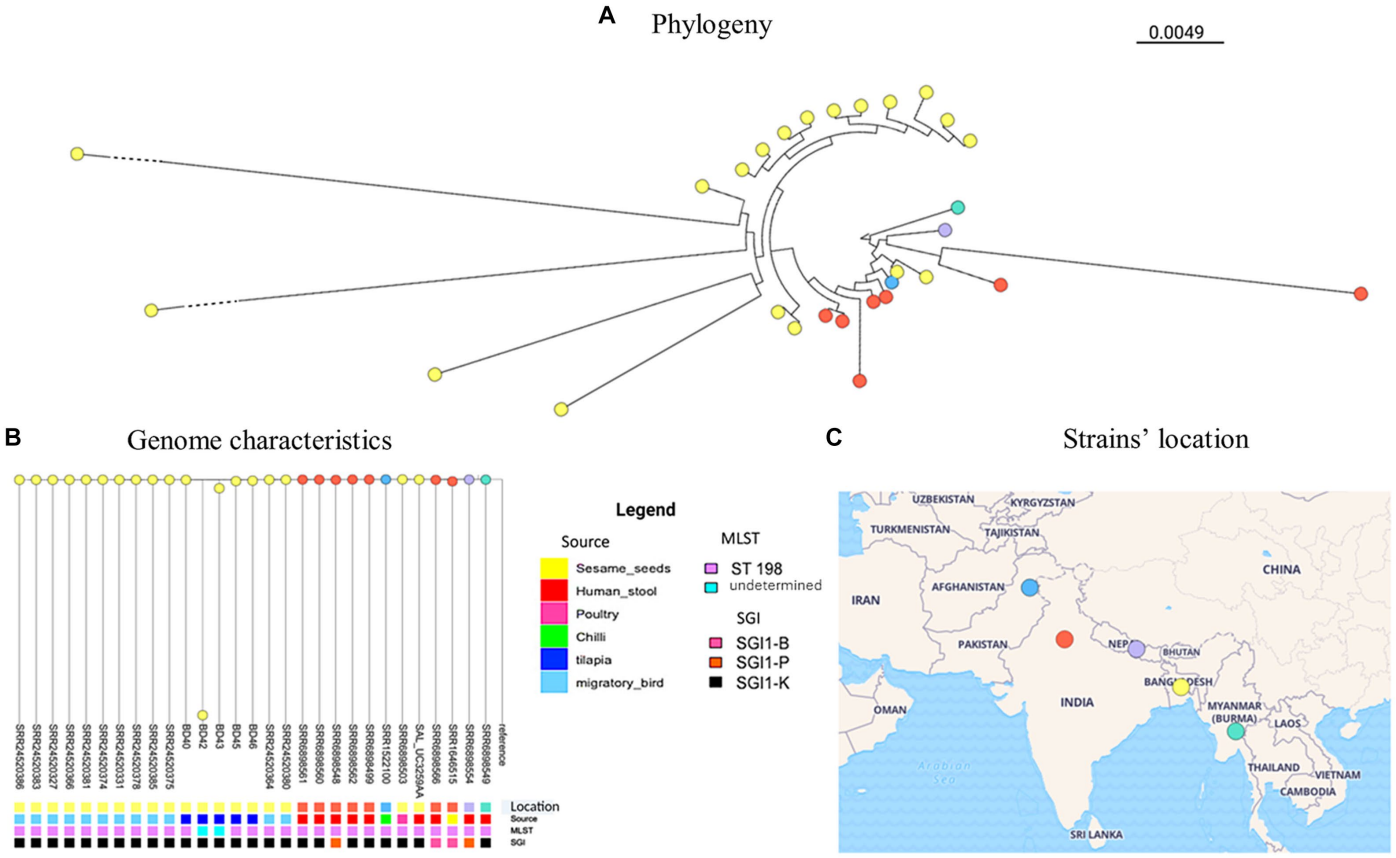
*S*. Kentucky phylogeny **(A)** including publicly available genome characteristics of *S*. Kentucky **(B)** originating from Bangladesh and surrounding countries. The geographical location of strains included from each country is shown on the map **(C)**.

The phylogenetic analysis of the genomes of the five *S*. Kentucky isolates from this study along with publicly available genomes of *S*. Kentucky from Bangladesh and neighboring countries revealed different levels of genetic relatedness among the strains circulating in the region and most of the isolates in our study (Figure 1, Supplementary Table S3). Out of all the tilapia isolates, BD40 had the least number of SNPs compared with the non-tilapia isolates (e.g., 30 SNPs with a migratory bird isolate). BD42 shows the greatest number of SNPs compared with BD43 (5,306 SNPs) and all remaining isolates. This was followed by BD43. BD42 and BD43 (non-ST198) clustered together. The *Salmonella* isolates from tilapia isolates evolved in one phylogenetic cluster from a common ancestor with the migratory bird isolates (SRR24520380/64), which have then further diverged with the remaining 10 migratory birds’ isolates (see further details on pairwise matrix on *S*. Kentucky Supplementary Table S4).

## Discussion

In this study, we assessed the occurrence of *E. coli* and NTS and the AMR of such isolates obtained from tilapia and shrimp products at retail, to assess the potential food safety and health risk to consumers.

The high occurrences of *E. coli* in skin swabs, gill, muscle, and intestinal samples from tilapia and shrimp sold at two main wet markets in Dhaka, Bangladesh indicates that fecal pollution and cross-contamination were common at the markets. The findings of *E. coli* in intestinal samples suggest that the tilapia and shrimp were raised in aquatic systems with fecal pollution, as *E. coli* is not part of the normal intestinal flora in fish and shrimp (Mohamed Hatha et al., 2003; Duran and Marshall, 2005; Vu et al., 2018), which contrasts with livestock such as in poultry and pigs (Dang and Dalsgaard, 2012; AbuOun et al., 2020; Islam et al., 2023). *E. coli* in muscle tissue may indicate cross-contamination, e.g., from the fish surface, during collection of the muscle tissue with other tissues before analysis. *E. coli* may also have entered the muscle tissue, e.g., from the fish gut and surface; however, this seems only to occur under highly stressed environmental aquatic conditions (Dang and Dalsgaard, 2012). It should be noted that the likelihood and levels of fecal contamination at markets may show seasonal variations, e.g., as a result of flooding and decreased hygiene conditions at markets.

On the contrary, there is no apparent explanation of the frequent finding of *Salmonella* spp. in gill and skin samples and their isolation in muscle tissue and intestinal samples, but the two latter samples are less likely to be fecally contaminated, e.g., during handling of the tilapia at the markets as opposed to surface skin and gills. While fish do not normally have *Salmonella* as part of their natural flora, they can become passive carriers (Fernandes et al., 2018). Reporting of *Salmonella* spp. in different types of seafood is relatively common due to fecal cross-contamination during handling and processing, but different serovars have also been found in the skin, gills, muscle, intestine, and feces of live fish (Fernandes et al., 2018; Baniga et al., 2019; Hounmanou et al., 2020, 2022).

The five *Salmonella* Kentucky strains analyzed in this study carried the SGI1-K island; all had an In4-type class 1 integron that contained only one cassette array and an adjacent mercury resistance module. Other studies have also found such a type of class 1 integrons in *S*. Kentucky as well as in other serovars (Levings et al., 2007; Doublet et al., 2009). The SGI-K island harbored resistance genes such as *aac(6′)-Iaa, bla_TEM-1B_, sul1*, *tet*(*A*), *aac(3)-Id*, and *aadA7* that confer resistance to aminoglycosides, tetracycline, sulphonamide, and narrow spectrum beta-lactam antibiotics. The set of genes present in these strains is, however, different from that reported in a previous study (Hawkey et al., 2019); for instance, the presence of *bla*_TEM1_ on the SGI-K is uncommon. This may be attributed to the plasticity of the genomes that allows for the loss or gain of segments by homologous recombination and which is responsible for the development of several variants of SGI1 in *Salmonella* (Hawkey et al., 2019; Jibril et al., 2021). Moreover, on the SGI-K island of the strains, the resistance genes were located on the IS6 transposon as were also found in recent poultry isolates in Nigeria (Jibril et al., 2021), showing the potential distribution of resistance through these islands via various mobile elements.

The identification of SPI-1, SPI-2, and SPI-5 in *S*. Kentucky, as well as an IncQ1 plasmid, further highlights the genetic diversity and potential for horizontal gene transfer among these isolates. Notably, three of the isolates were found to belong to ST198. Of particular interest are the strains BD42 and BD43, which branched separately on the phylogenetic tree. This finding suggests the presence of distinct genetic lineages or recent evolutionary events contributing to the diversification of *S*. Kentucky strains in the study area as can already be observed in the distinctive SPIs compared to the other three strains.

When comparing our strains to publicly available genomes of *S*. Kentucky from countries around Bangladesh, we observed different levels of genetic relatedness among the strains circulating in the region and most of the isolates in our study. This suggests a potential regional dissemination of *S*. Kentucky strains and emphasizes the importance of continued surveillance and molecular epidemiological studies to monitor the spread and evolution of this pathogen. Since *S*. Kentucky is rarely isolated from fish, these findings support fecal contamination from human or animal origin at the wet markets where the samples were obtained. Nevertheless, fish can be asymptomatic carriers, where *Salmonella* spp. have been isolated from surface tissues, muscle, and intestine (Fernandes et al., 2018; Baniga et al., 2019; Hounmanou et al., 2020, 2022). In Bangladesh, *S*. Kentucky has also been reported in poultry and migratory birds (Card et al., 2023), which show close relatedness with the isolates from this study.

## Conclusion

Our study showed a high level of fecal contamination with common findings of commensal *E. coli* in different sample types of tilapia and shrimp sold at two main wet markets in Dhaka. Together with the occurrence of *Salmonella* spp. in several products, e.g., *S*. Kentucky ST198, a well-known human pathogen, stresses the need to improve hygienic practices and sanitation standards at markets as well as in people’s homes to improve food safety and protect consumer health. Transmission of AMR bacteria to humans can occur directly through the consumption of or contact with contaminated products, posing a significant public health concern. Further genomic epidemiological analysis and disease burden estimations are needed in Bangladesh to assess the contribution of seafood to the overall occurrence of human salmonellosis as well as AMR problems in humans.

## Scope statement

Retail wet markets in low- and middle-income countries are often reported to have inadequate sanitation resulting in fecal microbial contamination. Such contamination of sold produce with antimicrobial resistant (AMR) bacteria can be a potential risk to public health. In this study, we conducted a pilot genomic surveillance for antimicrobial resistant *E. coli* and nontyphoidal *Salmonella* spp. isolated from tilapia and shrimp products purchased at two largest wet markets in Dhaka using Oxford nanopore sequencing. The study's results provide important insights into the prevalence and transmission of antimicrobial-resistant *E. coli* and *Salmonella* in tilapia and shrimp sold at wet markets. This has the potential to play a crucial role in efforts aimed at reducing the risk of transmitting fecal bacterial pathogens and antimicrobial resistance throughout the food chain.

## Data availability statement

The datasets generated and analyzed during the current study that support our findings are available in the National Center for Biotechnology Information (NCBI) repository at the following persistent web links: demultiplexed FastQ files for all 24 strains of *E. coli* and *Salmonella* spp. can be found under BioProject https://dataview.ncbi.nlm.nih.gov/object/PRJNA974206 PRJNA974206 with the corresponding BioSample accession numbers from https://dataview.ncbi.nlm.nih.gov/object/SAMN35176355 SAMN35176355 to https://dataview.ncbi.nlm.nih.gov/object/SAMN35176378 SAMN35176378 and Sequence Read Archive (SRA) accession numbers from https://dataview.ncbi.nlm.nih.gov/object/SRR24673188 SRR24673188 to https://dataview.ncbi.nlm.nih.gov/object/SRR24673211 SRR24673211.

## Ethics statement

Ethical approval was not required for the study involving animals in accordance with the local legislation and institutional requirements because the authors confirm that the ethical policies of the journal, as noted on the journal’s author guidelines page, have been adhered to. No ethical approval was required as no live animals were used in this study. The tissues were obtained from fish killed as part of routine commercial food production (i.e., sourced from wet markets).

## Author contributions

SR: Conceptualization, Formal analysis, Investigation, Methodology, Validation, Writing – original draft, Writing – review & editing. SH: Conceptualization, Formal analysis, Investigation, Methodology, Writing – original draft, Writing – review & editing. MSS: Methodology, Supervision, Writing – review & editing. FA: Investigation, Methodology, Writing – review & editing. LK: Conceptualization, Writing – review & editing. HMG: Data curation, Formal analysis, Writing – review & editing. AP: Conceptualization, Methodology, Writing – review & editing. RM-C: Writing – review & editing. YM-G-M: Data curation, Formal analysis, Writing – original draft, Writing – review & editing. AD: Formal analysis, Visualization, Writing – original draft. CVM: Conceptualization, Writing – review & editing. ZBB: Investigation, Writing – review & editing. MA-S: Conceptualization, Supervision, Writing – review & editing. DV-J: Methodology, Writing – review & editing. JD-D: Conceptualization, Data curation, Formal analysis, Investigation, Methodology, Supervision, Writing – original draft, Writing – review & editing.

## Footnotes

^1^https://github.com/nanoporetech/medaka

^2^https://pubmlst.org/

^3^https://github.com/tseemann/mlst

^4^https://pathogen.watch/

^6^https://github.com/tseemann/snippy

^7^https://microreact.org/
