# Supplementary material for: Nanopore sequencing for identification and characterization of antimicrobial-resistant Escherichia coli and Salmonella spp. from tilapia and shrimp sold at wet markets in Dhaka, Bangladesh
Source: Front Microbiol. 2024 Mar 7;15:1329620. doi: 10.3389/fmicb.2024.1329620 (PMC10956512; doi:10.3389/fmicb.2024.1329620)
Supplement: Supplementary file 1 [file Table_1.docx]

**Table S1 -** Metadata on sequenced strains and assembly statistics.

| **Isolate ID**  **Assembly** | **Species** | **Date of**  **isolation** | **Sample source** | **# contigs** | **Total length** | **Largest contig** | **GC (%)** | **N50** | **L50** |
| --- | --- | --- | --- | --- | --- | --- | --- | --- | --- |
| BD08.medaka | *E. coli* | 19/9/2021 | shrimp/muscle | 2 | 4897945 | 4804609 | 50.76 | 4804609 | 1 |
| BD09.medaka | *E. coli* | 19/9/2021 | shrimp/muscle | 1 | 4503495 | 4503495 | 50.83 | 4503495 | 1 |
| BD14.medaka | *E. coli* | 19/9/2021 | shrimp/muscle | 1 | 4649011 | 4649011 | 50.86 | 4649011 | 1 |
| BD01.medaka | *E. coli* | 31/10/2021 | shrimp/muscle | 1 | 4725736 | 4725736 | 50.79 | 4725736 | 1 |
| BD03.medaka | *E. coli* | 31/10/2021 | shrimp/muscle | 13 | 4762149 | 1507025 | 50.78 | 571494 | 3 |
| BD13.medaka | *E. coli* | 31/10/2021 | shrimp/muscle | 4 | 4747384 | 4695890 | 50.78 | 4695890 | 1 |
| BD23.medaka | *E. coli* | 31/10/2021 | shrimp/muscle | 1 | 4710044 | 4710044 | 50.91 | 4710044 | 1 |
| BD12.medaka | *E. coli* | 12/9/2021 | tilapia/skin | 2 | 4756431 | 4755807 | 50.72 | 4755807 | 1 |
| BD05.medaka | *E. coli* | 17/10/2021 | tilapia/skin | 109 | 3968775 | 141171 | 50.86 | 51615 | 25 |
| BD19.medaka | *E. coli* | 17/10/2021 | tilapia/skin | 2 | 4825005 | 4812094 | 50.69 | 4812094 | 1 |
| BD21.medaka | *E. coli* | 17/10/2021 | tilapia/skin | 1 | 4731366 | 4731366 | 50.81 | 4731366 | 1 |
| BD22.medaka | *E. coli* | 17/10/2021 | tilapia/skin | 11 | 4951226 | 1216723 | 50.71 | 1073802 | 3 |
| BD11.medaka | *E. coli* | 12/9/2021 | tilapia/muscle | 2 | 4709845 | 4690790 | 50.66 | 4690790 | 1 |
| BD17.medaka | *E. coli* | 17/10/2021 | tilapia/muscle | 4 | 5077851 | 4863496 | 50.84 | 4863496 | 1 |
| BD20.medaka | *E. coli* | 10/10/2021 | tilapia/muscle | 33 | 5029587 | 3529179 | 50.53 | 3529179 | 1 |
| BD40.medaka | *S. enterica* | 12/9/21 | tilapia/gills | 4 | 4884144 | 4848628 | 52.19 | 4848628 | 1 |
| BD41.medaka | *S. enterica* | 12/9/21 | tilapia/gills | 15 | 4807356 | 4711134 | 52.16 | 4711134 | 1 |
| BD43.medaka | *S. enterica* | 12/9/21 | tilapia/gills | 118 | 4830074 | 212041 | 52.28 | 73431 | 21 |
| BD25.medaka | *S. enterica* | 10/10/21 | tilapia/gills | 87 | 4774230 | 292647 | 52.14 | 90836 | 18 |
| BD35.medaka | *S. enterica* | 28/11/21 | tilapia/gills* | 7 | 4832090 | 2827907 | 51.96 | 2827907 | 1 |
| BD37.medaka | *S. enterica* | 28/11/21 | tilapia/gills* | 214 | 4703586 | 123284 | 51.92 | 30882 | 47 |
| BD42.medaka | *S. enterica* | 12/9/21 | tilapia/skin | 189 | 4924732 | 169017 | 52.05 | 40461 | 35 |
| BD45.medaka | *S. enterica* | 12/9/21 | tilapia/skin | 35 | 4887981 | 907969 | 52.23 | 409839 | 4 |
| BD46.medaka | *S. enterica* | 12/9/21 | tilapia/skin | 34 | 4887734 | 706397 | 52.13 | 237434 | 7 |
