# Supplementary material for: Nanopore sequencing for identification and characterization of antimicrobial-resistant Escherichia coli and Salmonella spp. from tilapia and shrimp sold at wet markets in Dhaka, Bangladesh
Source: Front Microbiol. 2024 Mar 7;15:1329620. doi: 10.3389/fmicb.2024.1329620 (PMC10956512; doi:10.3389/fmicb.2024.1329620)
Supplement: Supplementary file 3 [file Table_3.docx]

**Table S3-** *Salmonella* Kentucky genomes included in the phylogenetic analysis.

| **Biosample** | **ID** | **Country** | **Year** | **ST** | **Source** | **SGI** |
| --- | --- | --- | --- | --- | --- | --- |
| SAMN02846939 | SRR1646515 | India | 2010 | 198 | Sesame seeds | unknown |
| SAMN08784182 | SRR6898566 | India | 2010 | 198 | Human (stool) | SGI1-B |
| SAMN08784264 | SRR6898554 | Nepal | 2016 | 198 | Human (urine) | SGI1-P |
| SAMN08784224 | SRR6898503 | Bangladesh | 2009 | 198 | Layer poultry farms | SGI1-K |
| SAMN02874022 | SRR1522100 | Pakistan | 2014 | 198 | Red chilli powder | SGI1-K |
| SAMN08784228 | SRR6898499 | India | 2010 | 198 | Human (stool) | SGI1-K |
| SAMN08784229 | SRR6898562 | India | 2011 | 198 | Human | SGI1-K |
| SAMN08784261 | SRR6898548 | India | 2016 | 198 | Human (stool) | SGI1-P |
| SAMN08784230 | SRR6898561 | India | 2011 | 198 | Human (stool) | SGI1-K |
| SAMN08784231 | SRR6898560 | India | 2011 | 198 | Human (stool) | SGI1-K |
| SAMN08784262 | SRR6898549 | Myanmar | 2016 | 198 | Human (stool) | SGI1-K |
| SAMN35037656 | SRR24520386 | Bangladesh | 2018 | 198 | Migratory bird, *Aythya farina* (cloacal swab) | SGI1-K |
| SAMN35037657 | SRR24520375 | Bangladesh | 2018 | 198 | Migratory bird, Missing (cloacal swab) | SGI1-K |
| SAMN35037658 | SRR24520364 | Bangladesh | 2018 | 198 | Migratory bird, *Aythya farina* (cloacal swab) | SGI1-K |
| SAMN35037661 | SRR24520331 | Bangladesh | 2018 | 198 | Migratory bird, *Fulica atra* (cloacal swab) | SGI1-K |
| SAMN35037664 | SRR24520327 | Bangladesh | 2018 | 198 | Migratory bird, *Aythya farina* (cloacal swab) | SGI1-K |
| SAMN35037665 | SRR24520385 | Bangladesh | 2018 | 198 | Migratory bird, *Mareca strepera* (cloacal swab) | SGI1-K |
| SAMN35037667 | SRR24520383 | Bangladesh | 2018 | 198 | Migratory bird, *Aythya farina* (cloacal swab) | SGI1-K |
| SAMN35037669 | SRR24520381 | Bangladesh | 2018 | 198 | Migratory bird, *Fulica atra* (cloacal swab) | SGI1-K |
| SAMN35037670 | SRR24520380 | Bangladesh | 2018 | 198 | Migratory bird, *Aythya nyroca* (cloacal swab) | SGI1-K |
| SAMN35037672 | SRR24520378 | Bangladesh | 2018 | 198 | Migratory bird, *Mareca strepera* (cloacal swab) | SGI1-K |
| SAMN35037675 | SRR24520374 | Bangladesh | 2018 | 198 | Migratory bird, *Fulica atra* (cloacal swab) | SGI1-K |
| SAMN35037683 | SRR24520366 | Bangladesh | 2018 | 198 | Migratory bird, *Aythya nyroca* (cloacal swab) | SGI1-K |
| SAMN35176374 | BD40 | Bangladesh | 2021 | 198 | tilapia | SGI1-K |
| SAMN35176375 | BD42 | Bangladesh | 2021 | Und | tilapia | SGI1-K |
| SAMN35176370 | BD43 | Bangladesh | 2021 | Und | tilapia | SGI1-K |
| SAMN35176371 | BD45 | Bangladesh | 2021 | 198 | tilapia | SGI1-K |
| SAMN35176373 | BD46 | Bangladesh | 2021 | 198 | tilapia | SGI1-K |

Und: undetermined.
