# Supplementary material for: Nanopore sequencing for identification and characterization of antimicrobial-resistant Escherichia coli and Salmonella spp. from tilapia and shrimp sold at wet markets in Dhaka, Bangladesh
Source: Front Microbiol. 2024 Mar 7;15:1329620. doi: 10.3389/fmicb.2024.1329620 (PMC10956512; doi:10.3389/fmicb.2024.1329620)
Supplement: Supplementary file 5 [file Table_5.docx]

**Table S5 -** Antimicrobial resistance patterns in *E. coli* from fish and shrimp from wet markets.

| **Antimicrobial** | **Resistant (R)** | **Intermediate (I)** | **Susceptible (S)** |
| --- | --- | --- | --- |
| Chloramphenicol (CHL, 30 µg) | 0% (0/16) | 0% (0/16) | 100.00% (16/16) |
| Trimethoprim/sulphamethoxazole (SXT, 1.25/23.75/ µg) | 0% (0/16) | 0% (0/16) | 100.00% (16/16) |
| Ciprofloxacin (CIP, 5 µg) | 0% (0/16) | 6.25% (1/16) | 93.75% (15/16) |
| Cefepime (FEP, 30 µg) | 0% (0/16) | 0% (0/16) | 100.00% (16/16) |
| Meropenem (MEM, 10 µg) | 6.25% (1/16) | 0% (0/16) | 93.75% (15/16) |
| Ampicillin (AMP, 10 µg) | 25.00% (4/16) | 43.75% (7/16) | 31.25% (5/16) |
| Nalidixic acid (NAL, 30 µg) | 0% (0/16) | 18.75% (3/16) | 81.25% (13/16) |
| Norfloxacin (NOR, 10 µg) | 0% (0/16) | 0% (0/16) | 100.00% (16/16) |
| Cefoxitin (FOX, 30 µg) | 12.50% (2/16) | 0% (0/16) | 87.50% (14/16) |
| Gentamicin (GEN, 10 µg) | 6.25% (1/16) | 0% (0/16) | 93.75% (15/16) |
| Nitrofurantoin (NIT, 100 µg) | 18.75% (3/16) | 0% (0/16) | 81.25% (13/16) |
| Levofloxacin (LVX, 5 µg) | 0% (0/16) | 6.25% (1/16) | 93.75% (15/16) |
| Azithromycin (AZM, 15 µg) | 0% (0/16) | 0% (0/16) | 100.00% (16/16) |
| Cefuroxime sodium (CXM, 30 µg) | 6.25% (1/16) | 87.50% (14/16) | 6.25% (1/16) |
| Ceftriaxone (CRO, 30 µg) | 6.25% (1/16) | 6.25% (1/16) | 87.50% (14/16) |
