# Supplementary material for: Nanopore sequencing for identification and characterization of antimicrobial-resistant Escherichia coli and Salmonella spp. from tilapia and shrimp sold at wet markets in Dhaka, Bangladesh
Source: Front Microbiol. 2024 Mar 7;15:1329620. doi: 10.3389/fmicb.2024.1329620 (PMC10956512; doi:10.3389/fmicb.2024.1329620)
Supplement: Supplementary file 6 [file Table_6.docx]

**Table S6 -** Antimicrobial resistance patterns in *Salmonella* spp. from fish and shrimp from wet markets.

| **Antimicrobial** | **Resistant (R)** | **Intermediate (I)** | **Susceptible (S)** |
| --- | --- | --- | --- |
| Chloramphenicol (CHL, 30 µg) | 0% (0/14) | 0% (0/14) | 100.00% (14/14) |
| Trimethoprim/sulphamethoxazole (SXT, 1.25/23.75/ µg) | 0% (0/14) | 0% (0/14) | 100.00% (14/14) |
| Ciprofloxacin (CIP, 5 µg) | 35.71% (5/14) | 21.42% (3/14) | 42.86% (6/14) |
| Cefepime (FEP, 30 µg) | 0% (0/14) | 0% (0/14) | 100.00% (14/14) |
| Meropenem (MEM, 10 µg) | 0% (0/14) | 0% (0/14) | 100.00% (14/14) |
| Ampicillin (AMP, 10 µg) | 50.00% (7/14) | 0% (0/14) | 50.00% (7/14) |
| Nalidixic acid (NAL, 30 µg) | 35.71% (5/14) | 7.14% (1/14) | 57.14% (8/14) |
| Norfloxacin (NOR, 10 µg) | 28.57% (4/14) | 0% (0/14) | 71.43% (10/14) |
| Cefoxitin (FOX, 30 µg) | 21.42% (3/14) | 0% (0/14) | 78.57% (11/14) |
| Gentamicin (GEN, 10 µg) | 42.86% (6/14) | 0% (0/14) | 57.14% (8/14) |
| Nitrofurantoin (NIT, 100 µg) | 0% (0/14) | 0% (0/14) | 100.00% (14/14) |
| Levofloxacin (LVX, 5 µg) | 35.71% (5/14) | 0% (0/14) | 64.29% (9/14) |
| Azithromycin (AZM, 15 µg) | 0% (0/14) | 0% (0/14) | 100.00% (14/14) |
| Cefuroxime sodium (CXM, 30 µg) | 0% (0/14) | 57.14% (8/14) | 42.86% (6/14) |
| Ceftriaxone (CRO, 30 µg) | 7.14% (1/14) | 7.14% (1/14) | 85.71% (12/14) |
